# Mechanisms of Chitosan Nanoparticles in the Regulation of Cold Stress Resistance in Banana Plants

**DOI:** 10.3390/nano11102670

**Published:** 2021-10-11

**Authors:** Anbang Wang, Jingyang Li, Arwa Abdulkreem AL-Huqail, Mohammad S. AL-Harbi, Esmat F. Ali, Jiashui Wang, Zheli Ding, Saudi A. Rekaby, Adel M. Ghoneim, Mamdouh A. Eissa

**Affiliations:** 1 Hainan Banana Healthy Seedling Propagation Engineering Research Center, Haikou Experimental Station, Chinese Academy of Tropical Agricultural Sciences (CATAS), Haikou 571101, China; wanganbang011@126.com (A.W.); jingyanglee@163.com (J.L.); jiashuiwang@126.com (J.W.); 2 Department of Biology, College of Science, Princess Nourah Bint Abdulrahman University, Riyadh 13324-8824, Saudi Arabia; aaalhuqail@pnu.edu.sa; 3 Department of Biology, College of Science, Taif University, Taif 21944, Saudi Arabia; mharbi@tu.edu.sa (M.S.A.-H.); a.esmat@tu.edu.sa (E.F.A.); 4 Department of Soils and Water, Faculty of Agriculture, Al-Azhar University (Assiut Branch), Assiut 71524, Egypt; saudirekaby.4419@azhar.edu.eg; 5 Agricultural Research Center, Field Crops Research Institute, Giza 12112, Egypt; adelrrtc.ghoneim@gmail.com; 6 Department of Soils and Water, Faculty of Agriculture, Assiut University, Assiut 71526, Egypt

**Keywords:** chitosan nanoparticles, chilling tolerance, antioxidant enzyme activities, membrane damage traits

## Abstract

Exposure of banana plants, one of the most important tropical and subtropical plants, to low temperatures causes a severe drop in productivity, as they are sensitive to cold and do not have a strong defense system against chilling. Therefore, this study aimed to improve the growth and resistance to cold stress of banana plants using foliar treatments of chitosan nanoparticles (CH-NPs). CH-NPs produced by nanotechnology have been used to enhance tolerance and plant growth under different abiotic stresses, e.g., salinity and drought; however, there is little information available about their effects on banana plants under cold stress. In this study, banana plants were sprayed with four concentrations of CH-NPs—i.e., 0, 100, 200, and 400 mg L^−1^ of deionized water—and a group that had not been cold stressed or undergone CH-NP treatment was used as control. Banana plants (*Musa acuminata* var. Baxi) were grown in a growth chamber and exposed to cold stress (5 °C for 72 h). Foliar application of CH-NPs caused significant increases (*p* < 0.05) in most of the growth parameters and in the nutrient content of the banana plants. Spraying banana plants with CH-NPs (400 mg L^−1^) increased the fresh and dry weights by 14 and 41%, respectively, compared to the control. A positive correlation was found between the foliar application of CH-NPs, on the one hand, and photosynthesis pigments and antioxidant enzyme activities on the other. Spraying banana plants with CH-NPs decreased malondialdehyde (MDA) and reactive oxygen species (ROS), i.e., hydrogen peroxide (H_2_O_2_), hydroxyl radicals (^•^OH), and superoxide anions (O_2_^•−^). CH-NPs (400 mg L^−1^) decreased MDA, H_2_O_2_, ^•^OH, and O_2_^•−^ by 33, 33, 40, and 48%, respectively, compared to the unsprayed plants. We hypothesize that CH-NPs increase the efficiency of banana plants in the face of cold stress by reducing the accumulation of reactive oxygen species and, in consequence, the degree of oxidative stress. The accumulation of osmoprotectants (soluble carbohydrates, proline, and amino acids) contributed to enhancing the cold stress tolerance in the banana plants. Foliar application of CH-NPs can be used as a sustainable and economically feasible approach to achieving cold stress tolerance.

## 1. Introduction

Banana plants (*Musa* spp) belong to the *Musaceae* family and are widely cultivated in the tropical and subtropical regions of the world due to their high nutritive value, as well as their economic importance [1]. Banana fruits are a basic commodity and one of the most important sources of food in both developed and developing countries as they are are rich in carbohydrates, vitamins, and protein in addition to having high nutrient content, notably potassium, phosphorus, and calcium [2]. Climate changes and the continuous increase in the world population have led to an increase in the demand for food [3,4]. Bananas are grown in a wide range of tropical and subtropical regions around the world but their growth is negatively affected by various biotic and abiotic stresses, especially those caused by climate changes [5,6]. Chilling is one of the most important phenomena accompanying the emergence of climate changes, and it is also one of the most important reasons limiting the expansion of banana cultivation to meet the increase in food demand [7,8,9].

Low temperatures during the growth period of fruit trees lead to many physiological disorders within the plant cells, which have a negative impact on the quality of the fruit and result in a reduction of their marketing values [9,10]. There are many factors that affect the expansion of banana cultivation in the world, the most important of which is cold stress, which may cause losses of up to more than 50% of the yield, and this problem is exacerbated under climatic changes [8]. Banana plants are considered sensitive to cold stress, and when temperatures drop below the critical point (11 °C), physiological disorders occur, and the sensitivity differs depending on the variety [7,11]. The physiological disorders caused by cold stress negatively affect plant growth, [7,11]. Cold stress reduces the photosynthesis rate and causes various forms of damage in the cell membrane [12]. Banana plants tolerate cold stress by increasing the compatible solutes; e.g., proline, soluble carbohydrates, and phenolic compounds [7,8]. Various materials have been used to increase cold stress resistance and reduce damage in economically important plants [8,13]. Li et al. [13] used chitosan to increase the cold stress resistance of tea plants (*Camellia sinensis*), and found that chitosan increased plant tolerance to chilling. Currently, a technological revolution is taking place through the use of nanomaterials to increase plant resistance in the face of different abiotic stresses; despite this, there is not much information available on the effect of CH-NPs in increasing the resistance of bananas to chilling stress.

Nanotechnology can be used to address many agricultural and environmental challenges, and it can also be used as an effective tool for energy and resource constraints, the sustainable use of resources, urbanization, and fertilizer management [14]. With an ever-increasing population, there is a need for higher crop yields and more efficient strategies for improving agricultural practices, and thus the use of nanomaterials in agricultural science is on the rise [15]. Indeed, nanoparticle (NP) compounds may have a significant impact on sustainable agriculture and the development of precision agriculture by maximizing agricultural output (i.e., crop yields) and minimizing inputs (i.e., fertilizers, pesticides, and herbicides) via control of environmental conditions and the application of proper management [16]. NPs have properties distinct from those of bulk materials due to their small size and larger surface area which, as a result, mean that their solubility and surface reactivity are higher [17,18,19,20].

Chitosan (CH), produced from the polysaccharide chitin, has many benefits as an eco-friendly polymer in agricultural, biomedical, and fodder production [12,21,22]. CH-NPs are used for many purposes due to their high permeability, eco-friendliness, safety for living organisms, low price, and high solubility and biodegradability [23]. Many recent studies have recommended using CH-NPs to increase the ability of crops and economically important plants to address different stress conditions, such salinity, drought, metal toxicity, and heat [24,25].

The role of chitosan oligosaccharide in increasing the cold stress resistance of tea plants (*Camellia sinensis*) was studied by Li et al. [13]. They found that chitosan increased the accumulation of compatible solutes and the antioxidant defense of tea plants. However, to the best of our knowledge, there are no studies investigating the role of CH-NPs in the fight of banana plants (*Musa* spp) against cold stress. Therefore, the current study investigated the effects of the foliar application of CH-NPs in minimizing the negative impacts of chilling in banana plants. We assumed that the use of CH-NPs would increase the cold stress in banana plants by upregulating the defense mechanisms.

## 2. Material and Methods

### 2.1. Plant Materials and Chitosan Nanoparticles (CH-NPs)

Banana plants of the Baxi variety (*Musa acuminata* var. Baxi) produced with a tissue culture technique were used in the current study [7]. The plants were bought from the National Research Centre. The plants were three months old (40 cm high, 10 leaves) and cultivated in a growing media consisting of sand and compost (3:1 *w/w*). Twenty uniform, healthy plants were selected for the current experiment. Chitosan was bought from Sigma-Aldrich (St. Louis, MO, USA) and converted into nanoparticles in the Nanotechnology and Advanced Materials Laboratory of the National Research Centre (ARC). The CH-NPs had 99% purity, a specific surface area of 50–80 m^2^ g^−1^, and a particle size less than 100 nm. Banana plants were sprayed with four different concentrations of nanoparticles, i.e., 0, 100, 200, and 400 mg L^−1^ of CH-NPs in deionized water.

### 2.2. Chilling Treatments

Chilling treatment was undertaken in a plant growth chamber (LGP-250E, Shanghai, China). The chamber had a volume of 250 L and the plant growth conditions were automatically controlled. The selected plants were placed in the plant growth chamber under normal growth conditions (25 ± 1 °C, 60 ± 5% relative humidity, 12 h photoperiod with a PPFD of 250 μmol/(m^2^·s)). The plants were supplied with water and nutrient solution (50% Hoagland nutrient solution) [26]. The NP solutions used were prepared from a 1 g L^−1^ CH-NP stock solution: 1% (*w/v*) CH-NP solution was first prepared in 5% acetic acid (*v/v*) and, after stirring, the solution was diluted to 1:10 with deionized water. The final concentrations of 100, 200, and 400 mg L^−1^ were achieved by diluting the stock solution in deionized water. The plants in each treatment were sprayed with 250 mL of CH-NP solution containing Tween-80 (40 mL L^−1^). Seedlings of banana plants were sprayed with four levels of CH-NPs, i.e., 0, 100, 200, and 400 mg L^−1^. The plants were sprayed once a day with 250 mL of CH-NP solution for three continuous days, then the temperature in the incubator was set at 5 °C for 72 h without changes in the other growth conditions. A group that had not been cold stressed or undergone CH-NP treatment was used as control.

### 2.3. Plant Samples Analysis

The photosynthetic pigments (chlorophyll a + b and carotenoids) were extracted from the fresh leaves with acetone (80%) and then measured using a modified method from a study by Lichtenthaler and Buschmann [27]. The procedure described by Bates et al. [28] was used to determine the free proline in the fresh leaves of banana plants. Free proline was extracted from the fully expanded fresh leaf (0.1 g) with 3% sulfosalicylic acid (*w/v*). A method modified from the work of Ainsworth and Gillespie [29] was used in the measurement of the total phenolic compounds. Phenolic compounds were extracted from the banana leaves using methyl alcohol (85%). Soluble carbohydrates were extracted with ethanol (80%) and the total soluble carbohydrates were measured using the anthrone reagent method [7,30]. Two grams of each dried plant sample was digested using a mixture of H_2_O_2_, Se, Li_2_SO_4_, and concentrated H_2_SO_4_ [31]. The nitrogen in the plant sample extracts was measured using the Kjeldahl distillation method [32]. The phosphorus (P), potassium (K), calcium (Ca), iron (Fe), manganese (Mn), zinc (Zn), and copper (Cu) in the digested plant samples were determined using an inductively coupled plasma emission ICP 6200 (ICP) [32]. Reactive oxygen species (ROS), i.e., hydrogen peroxide (H_2_O_2_), hydroxyl radicals (^•^OH), and superoxide anions (O_2_^•−^), were determined using the method described by Velikova et al. [33] and Kubiś [34]. Hydrogen peroxide (H_2_O_2_) was extracted from fresh banana leaves with trichloroacetic acid (5%), then the absorbance reading was determined at 390 nm using spectrophotometer [33]. Superoxide anions (O_2_^•−^) were extracted from the leaf cuts using the K-phosphate buffer method, then the optical density was recorded at 580 nm [34]. Malondialdehyde (MDA) was determined based on the method described by Madhava and Sresty [35]. A fresh leaf sample (0.5 g) was extracted with 5 mL of 0.1% trichloroacetic acid (TCA) and then centrifuged, and the supernatant was added to 1 mL of 0.5% thiobarbituric acid in 20% TCA. Malondialdehyde concentration was calculated from the extinction coefficient of 155 mM^−1^ cm^−1^ [35]. Hydroxyl radicals (OH−) were extracted from 0.5 g of fresh sample with 10 mM Na phosphate buffer (pH 7.4) and 1% TBA, and then checked using a spectrophotometer at 550 nm [36]. The superoxide dismutase (SOD) activity was measured in 0.1 mM EDTA, sodium carbonate buffer (pH 10.2), epinephrine, and enzymatic extract, as described by Abeed et al. [37]. The peroxidase (POD) activity was measured spectrophotometrically following Abeed et al. [37].

### 2.4. Data Analysis

One-way ANOVA was used to test the significance of differences between the treatments. Means of treatments were compared using Tukey’s test at a 5% level of probability. The results in the figures and tables are shown as means (±SD, *n* = 5). All the statistical analyses were run in SPSS 17.0 (SPSS, Chicago, IL, USA).

## 3. Results

### 3.1. Effect of CH-NPs on the Growth and Nutrient Content of Banana Plants

**Figure 1:**
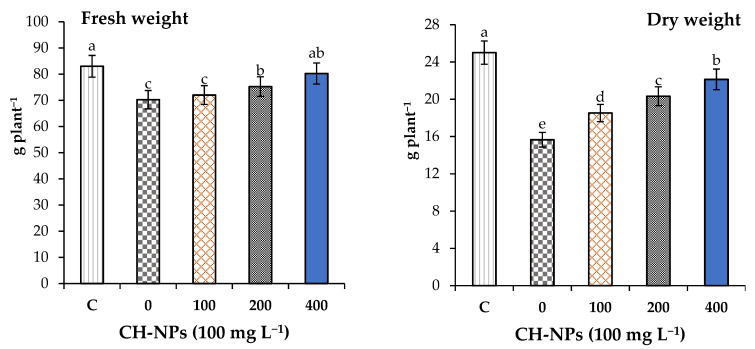
Effect of chitosan nanoparticles (CH-NPs) on the fresh and dry weights of banana plants exposed to cold stress (5 °C for 72 h). C = control treatment without cold stress and without CH-NP amendment Different letters refer to significant differences between treatments according to Tukey’s test at *p* < 0.05. Each value is the mean of five replicates (mean ± SD, *n* = 5).

Cold stress significantly reduced the growth of banana plants compared to the control treatment (C) (Figure 1). In this study, the foliar application of CH-NPs induced significant increases (*p* < 0.05) in the fresh and dry weights of banana plants under cold stress when compared to the unsprayed plants. The fresh and dry weights of banana plants treated with CH-NPs (100 mg L^−1^) were increased by 3 and 18%, respectively, when compared to the unsprayed plants, and these increases amounted to 7 and 30% in the case of the 200 mg L^−1^ treatment. Spraying banana plants exposed to cold stress with the highest concentration of CH-NPs (400 mg L^−1^) caused remarkable and significant increases in the fresh and dry weights, with increases of 14 and 41%, respectively, compared to the untreated plants.

The addition of CH-NPs to banana plants under cold stress significantly (*p* < 0.05) increased the nutrient content in the leaf tissue compared to the unsprayed plants (Table 1). Significant increases in N, P, K, Ca, and Fe were observed in CH-NP-treated plants (*p* < 0.05), while no alterations were observed in Mn, Zn, or Cu (Table 1). Foliar treatment with CH-NPs (100 mg L^−1^) induced increases in the concentrations of N, P, K, Ca, and Fe of 13, 12, 28, 27, and 4%, respectively, compared to the unsprayed plants. In the case of the 200 mg L^−1^ treatment, these increases were 19, 8, 33, 33, and 4%, respectively. Spraying banana plants with the highest concentration of CH-NPs (400 mg L^−1^) increased the N, P, K, Ca, and Fe by 19, 12, 44, 40, and 5%, respectively, compared to the untreated plants.

### 3.2. Effect of CH-NPs on the Secondary and Osmo-Metabolic Compounds

Cold stress increased the studied biochemical traits compared to the normal growth condaition. The foliar application of CH-NPs clearly increased the biochemical traits in the leaf tissue of banana plants grown under cold stress in comparison to the untreated plants (Table 2). Foliar application of all the studied concentrations of CH-NPs significantly enhanced the accumulation of the total phenolic compunds, soluble carbohydrates, proline, and amino acids in the leaf tissue of the banana plants grown under cold stress. Spraying banana plants with 100 mg L^−1^ of CH-NPs increased the total phenolic compounds, soluble carbohydrates, proline, and amino acids by 41, 31, 25, and 42%, respectively, compared to the unsprayed plants. Spraying Baxi banana plants with 200 mg L^−1^ of CH-NPs increased the total phenolic compounds, soluble carbohydrates, proline, and amino acids by 69, 33, 37, and 62%, respectively, compared to the control. Spraying banana plants with 400 mg L^−1^ of CH-NPs increased the phenolic compounds, soluble carbohydrates, proline, and amino acids by 79, 43, 50, and 112%, respectively, compared to the unsprayed plants. CH-NPs at all the studied concentrations increased the secondary and osmo-metabolic compounds in the leaves of the banana plants exposed to cold stress (5 °C for 72 h).

### 3.3. Effect of CH-NPs on the Photosynthesis Pigments and Antioxidant Enzyme Activities

**Figure 2:**
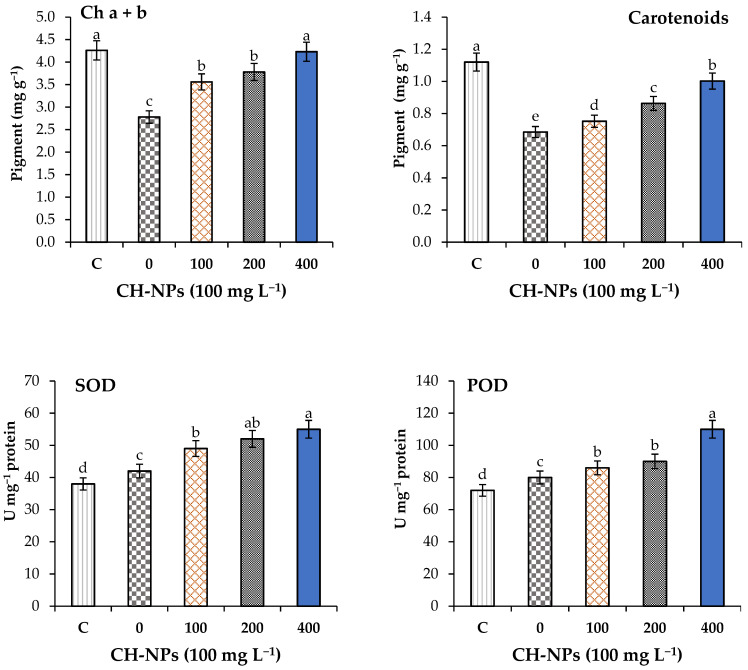
Effect of chitosan nanoparticles (CH-NPs) on photosynthesis pigments (Chl (a + b) and carotenoids) and antioxidant enzymes superoxide dismutase (SOD) and peroxidase (POD) of banana plants exposed to cold stress (5 °C for 72 h). C = control treatment without cold stress and without CH-NPs amendment. Different letters refer to significant differences between treatments according to Tukey test at *p* < 0.05. Each value is the mean of five replicates (mean ± SD, *n* = 5).

Spraying banana plants with CH-NPs significantly (*p* < 0.05) increased the photosynthesis pigments and antioxidant enzyme activities (Figure 2). Foliar application of CH-NPs significantly enhanced the carotenoids and total chlorophyll (a + b) in the leaf tissue of banana plants grown under cold stress. Spraying banana plants with 100 mg L^−1^ of CH-NPs increased the carotenoids and chl (a + b) by 10 and 28%, respectively, compared to the unsprayed plants. Spraying banana plants with 200 mg L^−1^ of CH-NPs increased the carotenoids and chl (a + b) by 26 and 36%, respectively, compared to the unsprayed plants. Spraying banana plants with 400 mg L^−1^ of CH-NPs increased the carotenoids and chl (a + b) by 46 and 52%, respectively, compared to the unsprayed plants. Foliar application of chitosan nanoparticles (CH-NPs) significantly enhanced the peroxidase (POD) and superoxide dismutase (SOD) in the leaf tissue of banana plants grown under cold stress.

Spraying banana plants with 100 mg L^−1^ of CH-NPs increased the peroxidase (POD) and superoxide dismutase (SOD) by 8 and 17%, respectively, compared to the unsprayed plants. Spraying banana plants with 200 mg L^−1^ of CH-NPs increased the peroxidase (POD) and superoxide dismutase (SOD) by 13 and 24%, respectively, compared to the untreated plants. Spraying banana plants with 400 mg L^−1^ of CH-NPs increased the peroxidase (POD) and superoxide dismutase (SOD) by 38 and 31%, respectively, compared to the control. CH-NPs at all the studied concentrations significantly (*p* < 0.05) increased photosynthesis pigments and antioxidant enzyme activities in the leaves of banana plants exposed to cold stress (5 °C for 72 h).

### 3.4. Effects of CH-NPs on the Stress Markers and Membrane Damage Traits

**Figure 3:**
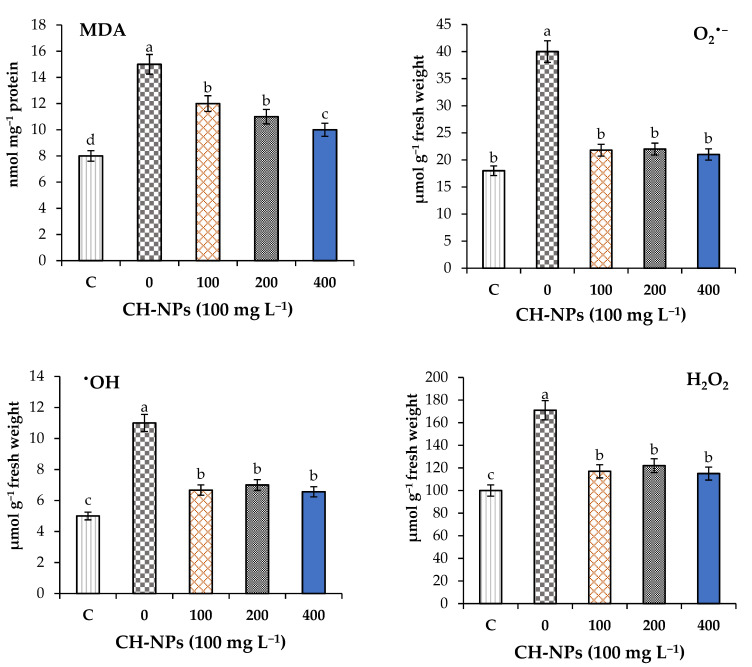
Effects of chitosan nanoparticles (CH-NPs) on malondialdehyde (MDA) and reactive oxygen species (superoxide anions (O_2_^•^^−^), hydroxyl radicals (^•^OH), and hydrogen peroxide (H_2_O_2_)). C = control treatment without cold stress and without CH-NP amendment. Different letters refer to significant differences between treatments according to Tukey’s test at *p* < 0.05. Each value is the mean of five replicates (mean ± SD, *n* = 5).

Reactive oxygen species (ROS), i.e., hydrogen peroxide (H_2_O_2_), hydroxyl radicals (^•^OH), and superoxide anions (O_2_^•−^), were determined and the data are shown in Figure 3. Furthermore, the malondialdehyde (MDA) was determined in order to explore the membrane damages, and the data are shown in the same figure.

The highest significant values (*p* < 0.05) for MDA and ROS were recorded in banana plants exposed to cold stress (5 °C for 72 h) without the addition of CH-NPs. Foliar application of chitosan nanoparticles (CH-NPs) significantly decreased MDA and ROS in the leaf tissue of banana plants grown under cold stress. Spraying banana plants with 100 mg L^−1^ of CH-NPs decreased the MDA, H_2_O_2_, ^•^OH, and O_2_^•−^ by 20, 32, 39, and 46%, respectively, compared to the unsprayed plants. Spraying banana plants with 200 mg L^−1^ of CH-NPs decreased the MDA, H_2_O_2_, ^•^OH, and O_2_^•−^ by 27, 29, 36, and 45%, respectively, compared to the untreated plants. Spraying banana plants with 400 mg L^−1^ of CH-NPs decreased the MDA, H_2_O_2_, ^•^OH, and O_2_^•−^ by 33, 33, 40, and 48%, respectively, compared to the unsprayed plants. In Figure 3, there is a clear trend indicating decreasing ROS and membrane damage traits in response to foliar application of all CH-NP concentrations.

## 4. Discussion

Climate change is considered to be a phenomenon that has recently increased in importance due to its negative effects on the growth of field crops and the quality of the crop yield, which directly affect the availability of food [38,39]. Low temperatures are one of the manifestations of these climatic changes that negatively affect the productivity of various crops, especially banana plants, as they are highly susceptible to cold stress [7,40,41]. Chilling harshly limits the physiological and biochemical processes in plant cells, causing leaf chlorosis, wilting, and even necrosis [42]. In the current study, the lowest significant growth values of the banana plants were recorded in the plants exposed to cold stress (5 °C for 72 h) without the addition of chitosan nanoparticles (CH-NPs). Cold stress occurs in banana plants when the temperature drops below the critical point (5–11 °C), which leads to various physiological disorders that negatively affect plant growth, and plants’ tolerance to cold stress varies according to the variety [7,11]. El-Mahdy et al. [7] studied the responses of two banana plant cultivars (Baxi and Williams) to cold stress (5 °C for 48 h) and found that cold stress reduced the growth of the two studied cultivars. They also confirmed that Baxi banana plants have more tolerance to chilling stress than the Williams variety. Banana varieties that have the ability to secrete compatible solutes and have a strong antioxidant defense are more tolerant to cold stress [7]. Furthermore, the protection of the photosynthesis system under cold stress conditions is an important mechanism that determines the degree of success of the plant’s resistance to chilling stress [7,8,13]. The protection of the fruit trees against chilling injury is crucial, and this can be done by developing efficient and practical techniques to reduce cold stress, particularly for banana plants. The findings of the current study confirm that chitosan nanoparticles (CH-NPs) are an effective tool in reducing the adverse effects of chilling in banana plants.

Nanoparticles (NPs) are an effective tool for promoting plant growth and enhancing stress tolerance [17,18,19,20,43]. NPs have properties distinct from those of bulk materials due to their small size and larger surface area which, as a result, mean that their surface reactivity is higher [17,18,19,20]. Spraying plants with nutrients, growth hormones, and important elements is an important agricultural process that stimulates plants to resist abiotic stress [13,18,24]. The application of CH-NPs enhances the growth of banana plants, as evidenced in this study by the increase in the fresh and dry weights. Once CH-NPs enter the leaf tissue, they can move and be transported to reach the centers of vital processes in the plant cell and become more effective in stimulating the plant’s resistance to stress [44,45,46,47].

**Figure 4:**
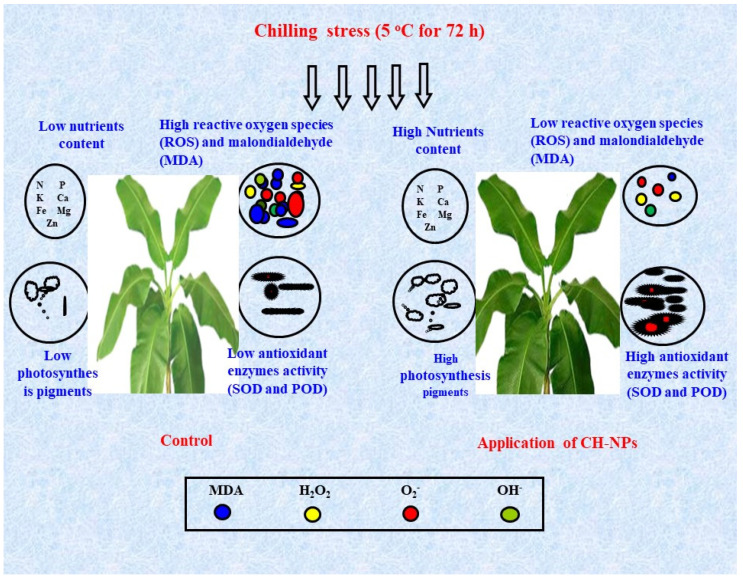
Schematic diagram exploring the roles of chitosan nanoparticles (CH-NPs) in enhancing the cold stress resistance of banana plants.

In the current study, we hypothesized that CH-NPs would increase the efficiency of banana plants in the face of cold stress by reducing the accumulation of reactive oxygen species and, in consequence, the degree of oxidative stress. The mechanisms behind this response seem to involve the stimulation of the activity of antioxidant enzymes and non-enzymatic antioxidants (phenolic compounds). The accumulation of osmoprotectants (soluble carbohydrates, proline, and amino acids), on the other hand, contributed to enhancing the plants’ resistance against chilling stress. In addition, the CH-NPs maintained a balance between nutrients and protection of photosynthesis pigments. The findings of the present study demonstrate that nutrient content, photosynthesis pigments, and antioxidant enzyme activities were significantly increased by the foliar application of CH-NPs; on the other hand, ROS and MDA were significantly decreased (Figure 4). The positive impacts of CH-NPs in reducing biotic and abiotic stresses have also been mentioned in other studies; e.g., Sen et al. [24], Zhang [25], and Sathiyabama and Manikandan [46].

The nutrients inside plants are considered one of the most important factors helping plants succeed in overcoming the harsh conditions that occur during cold stress [48,49]. The foliar application of CH-NPs enhanced the minerals status in the leaf tissue, e.g., Fe, Zn, Mn, P, Ca, N, K, and Mg. Chilling stress reduces the uptake of several nutrients, including N, P, and K [48,49]. The use of plant mineral nutrients is a potential option to achieve better crop growth and productivity and to alleviate the detrimental effects of chilling stress in a sustainable way [49,50]. Following the foliar application of CH-NPs, significant increases (*p* < 0.05) were recorded in most growth parameters and in the nutrient content of the banana plants exposed to cold stress (5 °C for 72 h). In particular, the increased magnesium and total nitrogen content contributed to increased chlorophyll content compared to the control [50,51,52,53,54,55,56,57].

## 5. Conclusions

The importance of the chilling problem is increasing with the increase in the demand for food in response to climatic changes. The current study clearly demonstrated that cold stress reduces growth, increases cell membrane injuries, and accumulates high levels of reactive oxygen species in leaf tissue. Spraying banana plants with chitosan nanoparticles (CH-NPs) at doses of 100–400 mg L^−1^ in water reduced the adverse effects of chilling. The positive effects of CH-NPs in enhancing chilling tolerance may be due to their role in reducing oxidative stress by enhancing the activity of antioxidant enzymes and increasing the concentration of osmoprotectant substances, which are used to control cell osmosis. Reducing the negative effects of cold stress with CH-NPs may be a sustainable and economically feasible approach to achieving abiotic stress tolerance. The use of CH-NPs to reduce chilling effects provides the opportunity to develop practical solutions for banana cultivation in places where cold stress occurs, especially following the emergence of various climatic changes in many agricultural production areas around the world.

## Figures and Tables

**Table 1:** Effect of chitosan nanoparticles (CH-NPs) on the elemental composition of leaves of banana plants exposed to cold stress (5 °C for 72 h).

CH-NPs mg L^−1^	N	P	K	Ca	Fe	Mn	Zn	Cu
	(g kg^−1^)	(g kg^−1^)	(g kg^−1^)	(g kg^−1^)	(mg kg^−1^)	(mg kg^−1^)	(mg kg^−1^)	(mg kg^−1^)
C	42 ± 4 a	2.8 ± 0.2 a	30 ± 2 a	23 ± 2 a	215 ± 12 a	152 ± 5 a	133 ± 6 a	57 ± 3 a
0	32 ± 2 d	2.5 ± 0.1 b	18 ± 1 d	15 ± 1 c	200 ± 4 c	150 ± 7 a	128 ± 5 a	56 ± 3 a
100	36 ± 3 c	2.8 ± 0.2 a	23 ± 2 c	19 ± 1 b	208 ± 5 b	154 ± 6 a	134 ± 7 a	57 ± 4 a
200	38 ± 2 b	2.7 ± 0.3 a	24 ± 3 c	20 ± 3 b	207 ± 6 b	152 ± 5 a	125 ± 4 a	56 ± 3 a
400	38 ± 3 b	2.8 ± 0.3 a	26 ± 3 b	21 ± 2 b	210 ± 8 a	155 ± 4 a	135 ± 6 a	58 ± 2 a

C = control treatment without cold stress and without CH-NP amendment. Different letters in the same column refer to significant differences between treatments according to Tukey’s test at *p* < 0.05. Each value is the mean of five replicates (mean ± SD, *n* = 5).

**Table 2:** Effect of chitosan nanoparticles (CH-NPs) on some biochemical traits in the leaves of banana plants exposed to cold stress (5 °C for 72 h).

CH-NPs (mg L^−1^)	Phenolic Compounds	Soluble Carbohydrates	Proline	Amino Acids
	(mg g^−1^)			
C	2.02 ± 0.8 d	22.52 ± 1.23 c	3.00 ± 0.07 e	22 ± 2 e
0	2.45 ± 0.05 c	26.57 ± 1.53 b	3.12 ± 0.09 d	26 ± 2 d
100	3.46 ± 0.06 b	34.68 ± 2.93 a	3.89 ± 0.13 c	37 ± 3 c
200	4.15 ± 0.09 a	35.23 ± 2.48 a	4.27 ± 0.10 b	42 ± 3 b
400	4.39 ± 0.04 a	38.12 ± 3.59 a	4.69 ± 0.12 a	55 ± 4 a

C = control treatment without cold stress and without CH-NP amendment. Different letters in the same column refer to significant differences between treatments according to Tukey’s test at *p* < 0.05. Each value is the mean of five replicates (mean ± SD, *n* = 5).

## Data Availability

The data presented in this study are available on request from the corresponding author.
